# Creating a standard inpatient opioid withdrawal protocol

**DOI:** 10.12688/mep.17533.1

**Published:** 2022-02-04

**Authors:** Ariana Abid, Steve Shoptaw, Michelle Bholat

**Affiliations:** 1 Family Medicine, UCLA ACCESS, Los Angeles, CA, USA; 2 Addiction Medicine, UCLA ACCESS, Los Angeles, CA, USA

**Keywords:** inpatient, opioid, withdrawal, buprenorphine, addiction, overdoses

## Abstract

**Background: **Despite safety and efficacy of medications for opioid use disorder, United States (US) hospitals face high health care costs when hospitalized patients with opioid use disorder (OUD) leave due to untreated opioid withdrawal.  Recent studies have concluded that evidence-based interventions for OUD like buprenorphine are underutilized by hospital services.

**Objective: **We developed a practical opioid withdrawal protocol utilizing buprenorphine and the Clinical Opiate Withdrawal Scale to address opioid withdrawal during inpatient treatment of a primary medical condition. We are currently implementing this protocol at the UCLA hospital in Santa Monica.

**Design: **The protocol includes order sets with appropriate and modifiable orders that can be submitted in the electronic medical record in order to deliver seamless care for opioid withdrawal. After the physician assesses the patient and initiates the protocol, nursing provides an essential role in continuing to monitor the patient’s level of withdrawal and administering the appropriate medications in response. Inpatient pharmacy is instrumental in monitoring medication administration, as well as calculating and providing dosages for orders on Day 2 and 3 of the protocol. Collaboration with case managers is essential for providing appropriate resources and ensuring a safe discharge.

**Conclusion:** Current challenges to widespread implementation of a standardized withdrawal protocol are discrepancies in addiction education across medical disciplines and inadequate outpatient access to buprenorphine providers and pharmacies that carry buprenorphine supplies.

## Introduction

Patients with substance use disorders are among the highest users of hospital services ^1^. With continually rising total nominal healthcare spending, increased overdoses and death of the current opioid epidemic, and costs associated with the COVID-19 pandemic ^2^, the very survival of hospitals may depend on developing cost-effective and evidence-based ways of providing care. Unfortunately, addiction-specific interventions for opioid use disorder during admission and at discharge are underused, and therefore a missed opportunity for saving costs and delivering quality care ^3^. Medication for opioid use disorder (MOUD) with methadone or buprenorphine, when compared to brief intervention and referral, has been shown to result in ^4^:

 - fewer relapses

 - decreased mortality rates

 - decreased acquisition of HIV infection

 - decreased criminal activity

 - increased rate of retention in rehabilitation programs

However, the approach of medication-assisted treatment has yet to adapted at the level of hospital care ^5^.

 Patients seen with opioid withdrawal typically suffer from the commonly overlooked diagnosis of opioid use disorder (OUD), and as a result, are seen most often at the hospital with complications such abscesses, cellulitis, endocarditis, osteomyelitis, septic arthritis, bacteremia, or history poly-substance use ^6^. Many prescribers struggle to diagnose and manage OUD in patients being treated with opioids for chronic pain. The treatment for both opioid withdrawal and OUD is buprenorphine, a partial opioid agonist with long-acting properties that act to minimize the “high” at the opioid receptors caused by other short-acting opioids ^7^.

## Proposed protocol

Our purpose is to suggest a practical and evidence-based protocol for hospitalists and other hospital care providers who are treating patients vulnerable to opioid withdrawal. We are currently implementing this protocol at UCLA hospital in Santa Monica, USA. The proposal was inspired by the legal allowance provided by Title 21 in the Code of Federal Regulations 1306.07c ^8^ regarding buprenorphine in the inpatient setting:

“This section is not intended to impose any limitations on a physician or authorized hospital staff to administer or dispense narcotic drugs in a hospital to maintain or detoxify a person as an incidental adjunct to medical or surgical treatment of conditions other than addiction, or to administer or dispense narcotic drugs to persons with intractable pain in which no relief or cure is possible or none has been found after reasonable efforts.”

**Figure 1.**
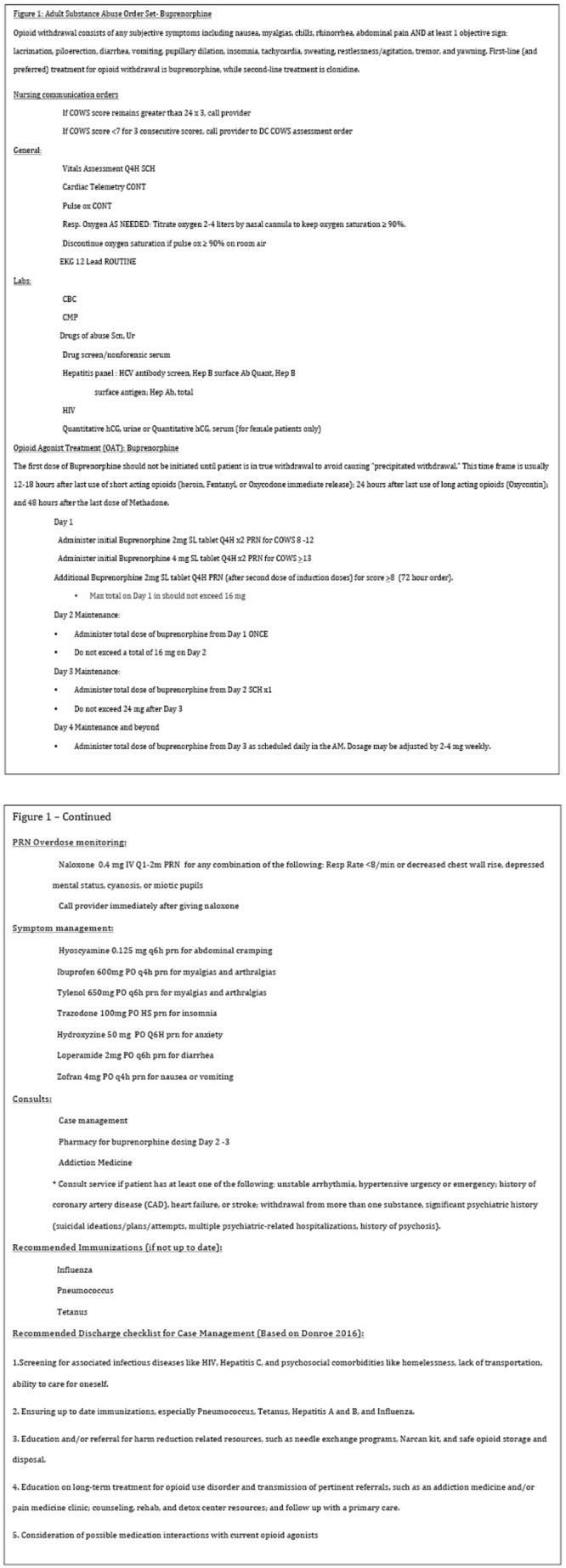


The protocol incorporates administration of buprenorphine guided by the use of a clinician tool called the Clinical Opioid Withdrawal Scale (COWS ^9^), which can be seen as parallel to the Clinical Intoxication Withdrawal Assessment (CIWA-AR; ^10^) for alcohol use disorder. Recommended order sets for treatment with buprenorphine are included ( Figure 1). An important note is that these orders can be adjusted based on feasible workflows and nursing availability of the institution; for example, additional buprenorphine can be provided within 1-3 hours as needed after the initial dose of buprenorphine if the patient complains of persistent, severe withdrawal symptoms ^11^.

The daily average maintenance dose ranges between 6–16 mg per day; however the provider must be mindful of individualizing care for each patient, which can be achieved by correlating doses with COWS scoring. Scores can be computed manually or through a flowsheet in the electronic medical record, where they can be easily monitored. Just as pharmacies assist with the dosing of vancomycin and warfarin, we feel that the inpatient pharmacist can also provide assistance in ensuring correct baseline dosing on day 2 and day 3 of the protocol, based on the addition of the doses received the previous day and ensuring the patient receives that specific amount for treatment consistency.

The first dose of buprenorphine should be provided when the patient is in moderate withdrawal or at least 12 hours after a short-acting opioid ^12^. The protocol is useful for providing these instructions to the healthcare provider. Patients should be very open about their use when offered buprenorphine as a relief, since the terrible symptoms of withdrawal are very uncomfortable and can be life threatening ^13^. In the event that a patient undergoes precipitated withdrawal after receiving the first dose of buprenorphine, the provider may provide supportive treatment in addition to clonidine or repeat buprenorphine 2 mg every one hour for four doses followed by administration of 8 mg for two doses (maximum 24 mg), until relief is achieved.

## Conclusion

Continuation of buprenorphine for long-term management in OUD is advised. This medication should be given in the form of buprenorphine/naloxone to discourage diversion. If the patient is unable to continue after discharge, the medication can be tapered, although this option is highly discouraged ^14^.

No study has established the optimal duration of MOUD, but it should be noted that patients with OUD are vulnerable to interruptions in their buprenorphine treatment ^15^ and have a high risk of relapse after stopping their maintenance medication ^16^. If the patient chooses not to continue buprenorphine, he/she must be educated on the risk of relapse and provided with referral for continued treatment. All patients with opioid use disorder should be discharged with naloxone and handouts on how to accept buprenorphine treatment ^17^.

Case management can play a key role in coordinating resources with outpatient providers, treatment centers, and/or living facilities based on insurance and social situation ^18^. Case managers should have an updated list of substance abuse treatment resources (including rehabilitation centers), and active buprenorphine providers to reference when coordinating a patient’s discharge.

 Current challenges to widespread implementation are misconceptions surrounding addiction treatment, discrepancies in addiction education across medical disciplines, and inadequate outpatient access to buprenorphine providers and pharmacies that carry buprenorphine supplies ^19^. However, these obstacles should not delay hospitals from establishing a standardized system for treating patients for opioid withdrawal, especially in the midst of the current opioid crisis. While education and expanding outpatient resources will remain an ongoing endeavor in the foreseeable future, opioid withdrawal must be treated with the same seriousness and standard of care as is generally done with any other medical problem for the duration of a patient’s inpatient stay.

### Ethical considerations

 Ethical approval was not sought/required as the protocol hasn’t been experimented yet, and no patient interaction or similar activity occurred.

## Data availability

No data are associated with this article.
