# Supplementary material for: Creating a standard inpatient opioid withdrawal protocol
Source: MedEdPublish (2016). 2022 Feb 4;12:7. [Version 1] doi: 10.12688/mep.17533.1 (PMC10366550; doi:10.12688/mep.17533.1)
Supplement: Peer review history

# Peer review: Reviewer response for version 1

**DOI:** 10.21956/mep.18808.r33622

**Published:** 2023-07-24

**Authors:** Jeremy Hayllar

**Affiliations:** 1 Metro North Mental Health, Alcohol and Drug Service, Brisbane, Queensland, Australia

Thanks for the opportunity to review this letter which makes an important case for improving the management of inpatients with opioid use disorder, treating withdrawals with 'protocolised' buprenorphine, viewed in the wider context of the terrible opioid epidemic in USA. Language and references are fine, save a couple of issues below.

 I agree with the premise that it is often the complications of OUD which bring the patient to hospital - I don't think "history poly-substance use" fits well with the other complications listed, and in the list perhaps ascending in severity *bacteremia *could follow *cellulitis* then *osteomyelitis, septic arthritis or endocarditis*. 

 In the same paragraph it could be argued that methadone can also be used to treat both opioid withdrawal and OUD - though I agree buprenorphine is to be preferred in this context.  Perhaps *current treatment...*

 Great to involve ward pharmacists to ensure patients receive appropriate doses of buprenorphine treatment. 

 In the final paragraph before the conclusion, consider bringing **precipitated** **withdrawal** to the previous sentence: *the terrible symptoms of precipitated withdrawal are very uncomfortable,* then **in the following sentence it is no longer needed.  

 In the first paragraph of the conclusion the availability of long-acting buprenorphine injections is changing the landscape of OUD treatment. Monthly or even weekly injections provides a larger window for discharged hospital patients to access community treatment? worth mentioning.

 Last sentence of 2nd paragraph of Conclusion - **access** better than *accept buprenorphine treatment.*

 The protocol is comprehensive, it walks the line between intervening too early risking ppt withdrawal and starting buprenorphine too late risking self-discharge because of untreated withdrawals.  

 A few more general questions for the authors to consider: 

 Diagnosis of OUD withdrawal is easy in retrospect, however there is some overlap with symptoms from a fever for example. Therefore recognising the condition is an important first step in the process, triggering use of COWS and starting treatment.

 How much experience do nursing staff require in administering the COWS? - while this is a validated tool how much influence does nursing experience have on the scores?

 Some inpatients may require urgent or less urgent surgery - perhaps drainage of a collection or more seriously valve replacement for endocarditis. Is it the view of the authors that buprenorphine treatment should be initiated regardless?  

 Some inpatients may seek pain management (I note ibuprofen in the protocol) should you mention that buprenorphine is expected to provide effective pain management?

 A small point - in our practice we generally initiate patients on buprenorphine/naloxone to avoid unnecessary changes in treatment say from buprenorphine tablet to bup/nal film in the community.

Is the rationale for the Open Letter provided in sufficient detail?

Yes

Does the article adequately reference differing views and opinions?

Yes

Are all factual statements correct, and are statements and arguments made adequately supported by citations?

Yes

Is the Open Letter written in accessible language?

Yes

Where applicable, are recommendations and next steps explained clearly for others to follow?

Yes

Reviewer Expertise:

Opioid Use Disorder

I confirm that I have read this submission and believe that I have an appropriate level of expertise to confirm that it is of an acceptable scientific standard.

# Peer review: Reviewer response for version 1

**DOI:** 10.21956/mep.18808.r31926

**Published:** 2022-05-09

**Authors:** Amer Raheemullah

**Affiliations:** 1 Department of Psychiatry and Behavioral Sciences, Stanford University School of Medicine, Palo Alto, CA, USA

This protocol is a symptom-triggered buprenorphine protocol for withdrawal management. The paper addresses how this protocol can operate similar to the commonly accepted symptom-triggered benzodiazepine protocols using CIWA for alcohol withdrawal. Providers and nurses are already habituated to treat withdrawal in this manner and look for concrete guidance on how to manage withdrawal. This protocol makes buprenorphine inductions accessible to hospitalists who may not otherwise feel comfortable carrying these out. Protocols like these are needed to increase access to buprenorphine to combat the opioid epidemic.

Is the rationale for the Open Letter provided in sufficient detail?

Yes

Does the article adequately reference differing views and opinions?

Yes

Are all factual statements correct, and are statements and arguments made adequately supported by citations?

Yes

Is the Open Letter written in accessible language?

Yes

Where applicable, are recommendations and next steps explained clearly for others to follow?

Yes

Reviewer Expertise:

Inpatient addiction medicine service director

I confirm that I have read this submission and believe that I have an appropriate level of expertise to confirm that it is of an acceptable scientific standard.
